# Retrospective empirical analysis of the success rate of inguinal hernia operations in outpatient and inpatient sectors: a cohort study

**DOI:** 10.1007/s10029-026-03601-1

**Published:** 2026-03-16

**Authors:** Julius Wiemschulte, Robert Messerle, Jonas Schreyögg

**Affiliations:** Hamburg Centre for Health Economics (HCHE), University of Hamburg, Esplanade 36, 20354 Hamburg, Germany

**Keywords:** Inguinal hernia, Hybrid-DRG, Outpatient treatment, Care sector, Surgical success

## Abstract

**Introduction:**

In Germany, the proportion of outpatient surgeries was low during the study period (2014–2019) and low by international standards. Less than 20% of inguinal hernias are treated on an outpatient basis. Hybrid DRGs are intended to promote outpatient treatment, but their impact on the quality of care and referral criteria has not been sufficiently investigated empirically.

**Methods:**

A retrospective analysis of routine data included 90,512 cases from 41 company health insurance funds, spanning 2014 to 2019. These were analysed descriptively in terms of care sector, age, and surgical procedure, as well as through two logistic regressions on reoperations and complications, including interaction effects.

**Results:**

The proportion of outpatient surgeries is already below 40 % in adults and continues to decline with increasing age. The choice of procedure differs significantly between sectors. The regressions explain only 3.3 % and 4 % of the variance, respectively, meaning that the variables have only a minor impact on the success of the surgery. Inpatient surgeries are associated with fewer reoperations and more complications, although the absolute effect size is small. The surgical procedures have a significant influence. There are no relevant interaction effects between the choice of sector and the other variables.

**Discussion:**

A sector-specific allocation based on the analysed parameters cannot be justified based on evidence. Since inpatient procedures do not show consistent superiority, there is no medical advantage over outpatient procedures. A cost-adjusted design of hybrid DRGs appears necessary to enable indication-appropriate procedure selection and to avoid potential misguided incentives that compromise the quality of care.

In recent years, the costs of the German healthcare system have increased sharply as a proportion of the country’s gross domestic product [1]. One frequently discussed measure for reducing costs is the shift to outpatient care. Inguinal hernia operations are often cited as an example of this, as they are considered to have a low complication rate and a high number of cases [2]. They are included in the AOP catalogue and may be performed on an outpatient or inpatient basis [3]. Outpatient treatment is generally preferred over inpatient treatment. Until 2023, the Health Appropriateness Evaluation Protocol (G-AEP) was used as an exclusion criterion for outpatient procedures, but this has since been replaced by contextual factors based on the Barthel Index and care level [4]. The final decision is based on the doctor’s assessment, allowing individual reasons for inpatient treatment to be considered even if they don’t meet the criteria.

According to the Federal Statistical Office, in 2023, almost one in every hundred inpatient operations was for the repair of an inguinal hernia (158,895 out of 16,531,491), making this operation the seventeenth most common [5]. The proportion of outpatient procedures is approximately 20% and has remained stable for over 15 years. Considering both inpatient and outpatient settings, the total number of inguinal hernia operations in Germany is estimated at over 250,000 per year [6]. This figure refers to operative treatment cases and excludes other types of hernia surgery, such as umbilical, incisional, or femoral hernias.

In an international comparison, the DACH countries rank at the bottom in terms of outpatient treatment. In Anglo-American and Scandinavian countries, the proportion of outpatient operations is over 80%. Some studies attribute this difference to remuneration incentives [7, 8]. In Germany, insufficient remuneration in the outpatient sector is viewed as a significant obstacle [6]. The remuneration for an outpatient hernia operation was only 25% of the inpatient fee. To provide an approximate reference point, a standard inpatient unilateral, uncomplicated inguinal hernia repair without abdominal wall reconstruction is reimbursed at €3,489.01 according to the aG-DRG catalogue, assuming an average length of stay and excluding the nursing care budget [9, 10]. For inpatient inguinal hernia operations alone, this results in a volume of over €560 million per year.

Despite the actual priority of outpatient over inpatient care and defined referral criteria (G-AEP and contextual factors), the goal of increasing outpatient care has not been achieved for years. With the introduction of hybrid DRGs in 2024, a uniform cross-sector remuneration system will be introduced to create financial incentives for outpatient care [11]. Comparable reforms in other countries have already led to a significant increase in outpatient care [12, 13]. Inguinal hernia operations were among the first procedures for which this new DRG remuneration model was introduced, as they are considered low-complication procedures with significant potential for financial savings.

Potential differences in quality between the care sectors are cited as key arguments against increased outpatient care [14]. Whether the frequently cited difference in treatment outcomes between outpatient and inpatient care is empirically accurate has not yet been sufficiently investigated in Germany. In particular, it remains questionable whether better outcomes are achieved in an inpatient setting when risk factors are present. The parallel use of various open and laparoscopic procedures further complicates the situation. Whether and which factors should be used to determine referral to the outpatient or inpatient sector has not yet been established on a sound empirical basis.

In contrast to the German sector selection, which focuses on functional abilities within the context of factors, the European guideline, based on the ASA score, emphasizes physical morbidity [15, 16]. In Germany, contextual factors are also permissible as direct (legal) justification for inpatient surgery in addition to individual medical assessment. In contrast, other countries with a high rate of outpatient surgery generally do not apply general exemption criteria for inpatient care; instead, they rely on individual justification by physicians [4, 14].

Against this background, an analysis of billing data for BKK-insured persons from 2014 to 2019 was conducted as part of the ‘Uniform, Sector-Equal Remuneration’ (ESV) project, which the G-BA Innovation Fund funded. The overall project was led by the Hamburg Centre for Health Economics (HCHE). It involved the German Hospital Institute (DKI), the Central Institute for Statutory Health Insurance Medical Care (Zi), the Technical University of Berlin, and the BKK Dachverband e. V. (funding code 01VSF19040). This work aimed to determine whether the choice of care sector (outpatient practice, outpatient care, or inpatient care in a hospital), the referral criteria used, and frequently discussed risk factors influence the success of surgery [6, 17, 18]. Demographic characteristics and comorbidities were taken into account. Gender was included due to anatomical differences, and age was considered about physical constitution and perioperative risks. Diseases such as obesity, COPD, diabetes mellitus, or Marfan syndrome affect intra-abdominal pressure and connective tissue structure and thus influence the course of the disease and healing. In addition, socio-psychological variables such as dementia, social support, and living conditions were considered, as they help determine the need for support and are utilized in healthcare practice as contextual factors for sector allocation. Reoperations and postoperative complications were regarded as indicators of failure. The analysis thus aims to make an empirical contribution to outpatient care by examining variables that influence sector selection.

The present analysis is based on routine data from 2014 to 2019 and therefore reflects the care situation before the introduction of hybrid DRGs in Germany. As such, it provides a baseline against which future changes in sector allocation and outcomes under the new reimbursement framework can be evaluated.

## Methods

**Fig. 1:**
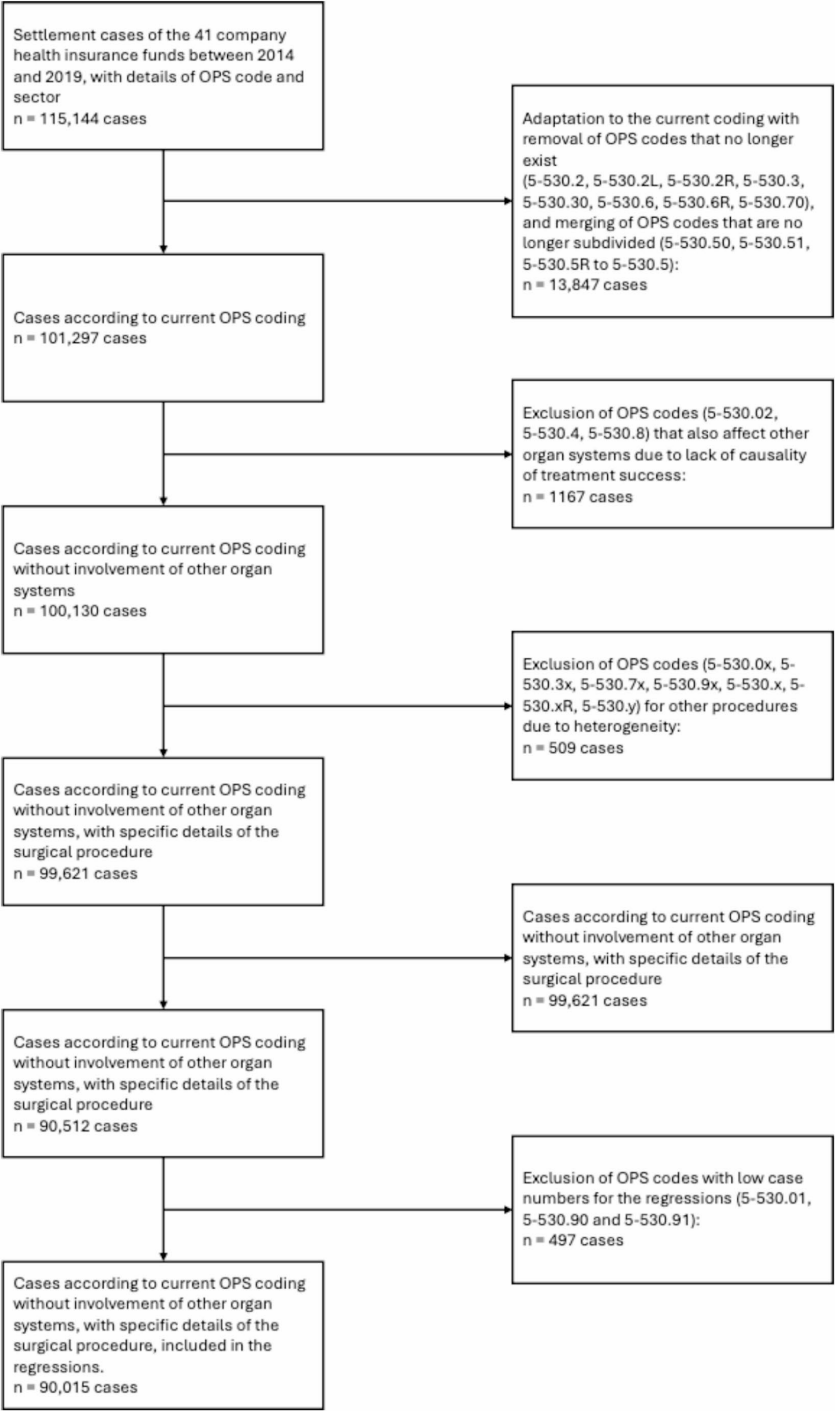
Data selection: inclusion and exclusion criteria based on OPS codes

This study on inguinal hernia operations is based on nationwide billing data from 41 company health insurance funds for the period from 2014 to 2019. All available cases for which the OPS code and treating sector information were available were included (Fig. 1).

Only adult patients were included in the analysis because the therapeutic concept for minors does not include watchful waiting and is therefore different.

A total of 90,512 cases were included in the analysis, and the data were initially evaluated descriptively for demographic factors. Subsequently, two logistic regressions were calculated, in which, in addition to main effects, interaction effects between the care sector and the other variables were modelled regarding the success of the operation. To ensure sufficient statistical power, OPS codes with too few cases were removed from the regression, resulting in 90,015 cases being included in the analysis. In the first model, the occurrence of a repeat surgical procedure within one year of the index operation served as the dependent variable. Both initial and repeat operations were considered as index operations.

In the second model, postoperative complications, such as infections and bleeding, within three months of the index operation were analysed. The definition was based on specific ICD codes (T81.0, T81.11, T81.2, T81.3, T81.4, T81.40, T81.41, T81.42, T85.78, T81.88). The selection of complications and the determination of the observation period are based on the WIdO’s quality assurance procedure, which utilizes routine data [19]. A causal relationship with the index operation is assumed for the periods. Deaths as a result of surgery were not analysed due to insufficient numbers.

## Results

Of the 90,512 cases, 81,208 were men (89.7%) and 9,304 were women (10.3%). Seventy-four thousand nine hundred ninety-one cases were treated as inpatients (82.9%), 5,993 as outpatients in a hospital (6.6%), and 9,528 as outpatients in a doctor’s office (10.5%). This means that the proportion of outpatient operations was around 17.1%. The rate of outpatient operations remained stagnant in each year of the study period (2014–2019). In each year, more than 80% of cases were treated as inpatients.

The gender distribution remained essentially constant during the study period, with no changes observed in the proportion of surgical procedures.

**Fig. 2:**
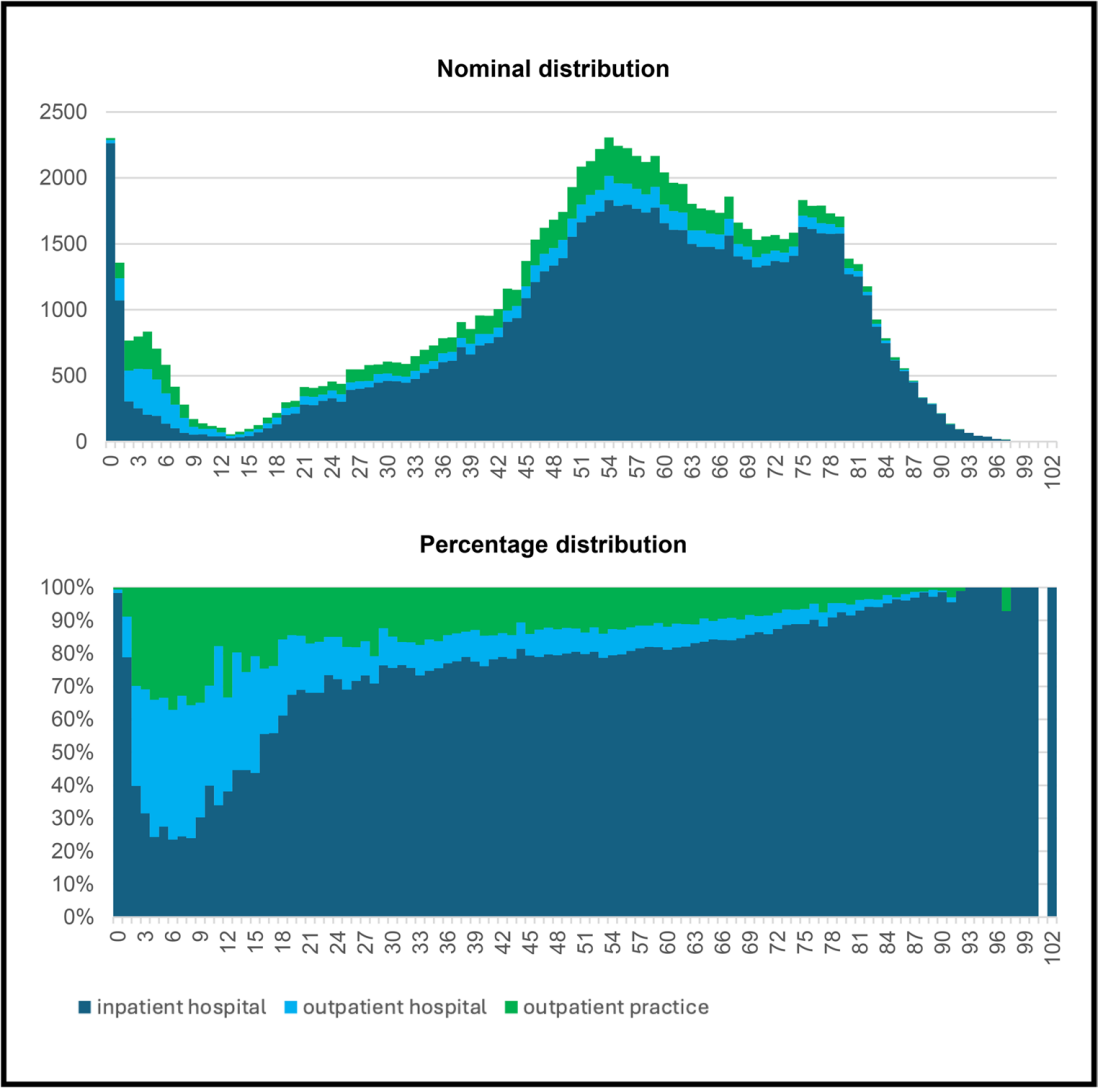
Sectoral breakdown by age

When examining the sectoral shares of care provision by age, apparent differences emerge (Fig. 2). The following analyses refer exclusively to adult patients. However, a supplementary look at minors reveals a contrast: around 75% of procedures on children aged 4 to 8 are performed on an outpatient basis, with this proportion falling significantly to below 40% by the age of 18.

The proportion of outpatient operations continues to decline with increasing age: it was 38.9% for 18-year-olds and 32.6% for 19-year-olds. From the age of 26, it remains below 30%, and from the age of 56, it remains below 20%.

A comparison of outpatient care locations reveals that the proportion of outpatient surgery in hospitals declines earlier in adulthood, from 23.2% (at 18 years) to 8.3% (at 28 years). In practice, on the other hand, the proportion remains stable at around 15% for an extended period and only declines gradually after the age of 35.

**Fig. 3:**
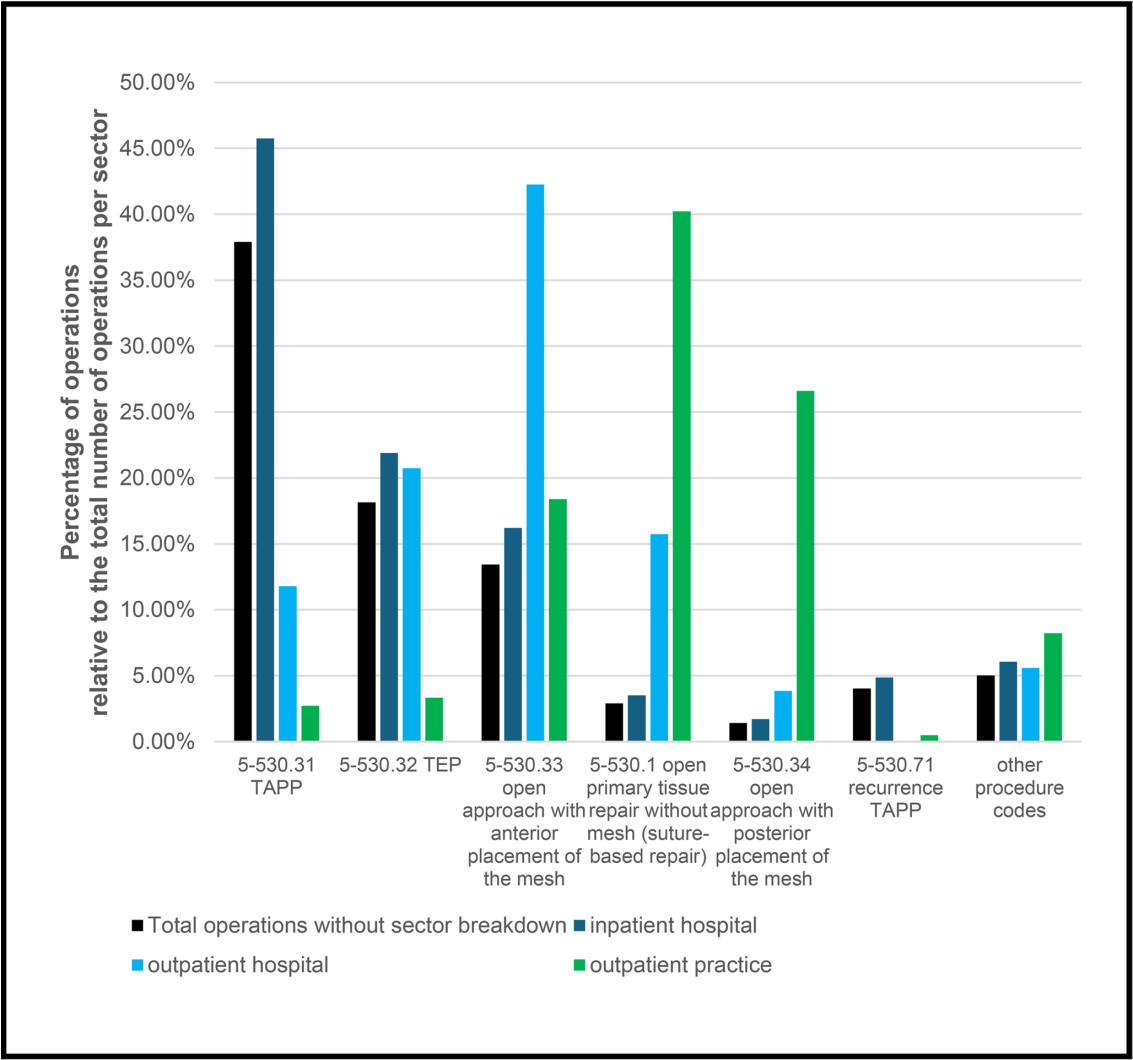
Percentage share of surgical procedures (OPS code) per sector

In addition, the cases could be differentiated according to the surgical methods used (Fig. 3). Overall, 9.4% of the procedures involved a recurrent OPS, although the primary procedure did not necessarily have to take place during the period under review. In 64.5% of cases, a laparoscopic procedure was used. Transabdominal preperitoneal mesh implantation (TAPP) was the most frequently chosen method at 39.0%, followed by totally extraperitoneal mesh implantation (TEP) at 19.9%.

Laparoscopic procedures were significantly more common in inpatient operations: TAPP in 45.8% and TEP in 21.9% of cases. In contrast, TAPP and TEP were only used in 32.6% of outpatient procedures in hospitals. Instead, open approach with anterior placement of the mesh dominated, accounting for 42.2%, and open surgical procedures with plastic closure were used in 15.7% of cases.

In practice, the proportion of laparoscopic procedures (TAPP and TEP) was particularly low at 6.0%. Instead, open surgical procedures were performed in 40.2% of cases and open approach with posterior placement of the mesh in 26.6% of cases. The latter were thus used significantly more frequently in practices than in hospitals.

**Fig. 4:**
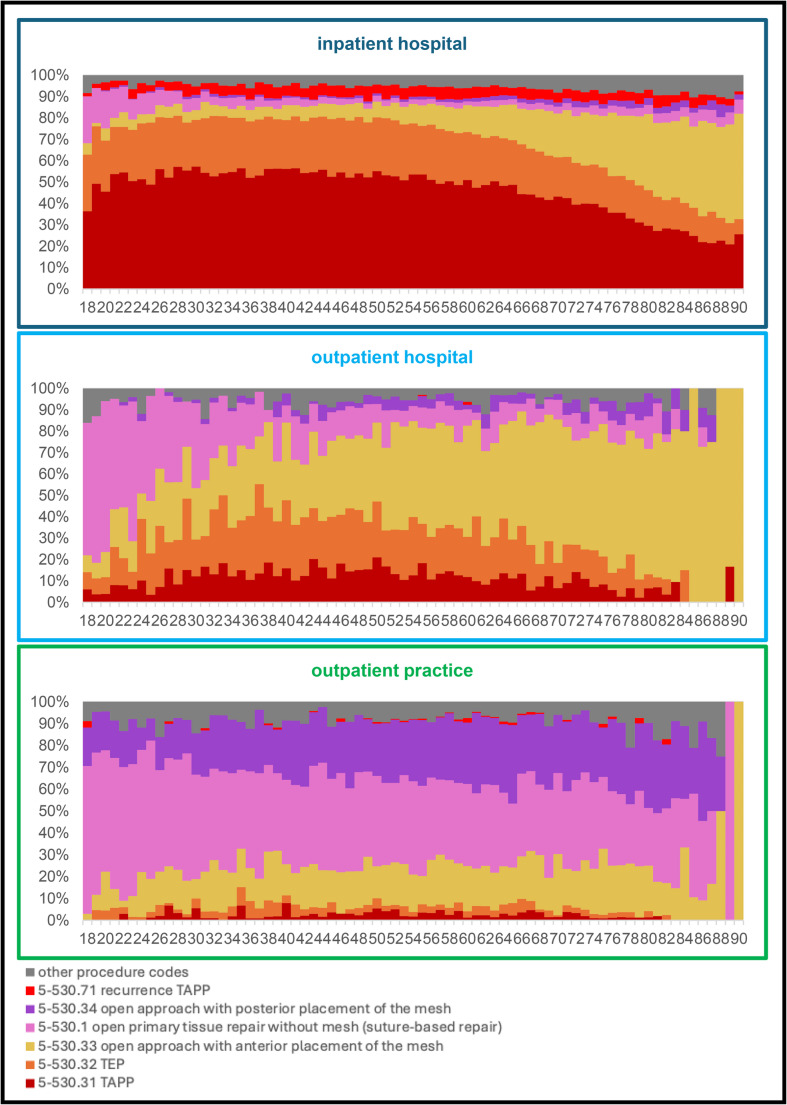
Distribution of surgical procedures by age (18–90 years) – broken down by healthcare sector

When OPS codes within the individual sectors are differentiated by age, clear trends emerge (Fig. 4). In the inpatient sector, the proportion of open approach with anterior placement of the mesh increases from the age of 55 at the expense of TAPP and TEP (from around 10 to around 35%). In outpatient surgery in hospitals, young adults are often operated on using open plastic surgery procedures. The proportion drops from around 70% at the age of 18 to around 20% at the age of 35. At the same time, the proportion of open approach with anterior placement of the mesh increases with age from about 10% at age 18 to about 70% at age 80. For surgeries in medical practices, the proportion remains relatively stable across all age groups.

The regression analyses included, among other factors, those listed in Table 1. Connective tissue disorders (such as Marfan syndrome or Ehlers-Danlos syndrome) could not be included as influencing factors due to the low number of cases. A test for multicollinearity revealed no evidence of problematic correlations.

**Table 1:** Logistic regressions for reoperations and complications

Variable	*n* = 90,015	Model for reoperations Odds ratio *p*-value, 95% confidence interval	Model for complications Odds ratio *p*-value; 95% confidence interval
**model quality**		reoperations = 774	complications = 4278
omnibus test		p-Wert < 2.2e-16***	p-Wert < 2.2e-16***
McFadden R^2^		0.033	0.041
discriminative ability – AUC		0.646	0.662
**care setting** comparison group inpatient hospital	74,991		
outpatient hospital	9528	1.416^*^ *p* = 0.017; [1.064; 1.883]	0.374^***^ *p* < 0,001; [0.312; 0.447]
outpatient practice	5993	1.328^*^ *p* = 0.034; [1.023; 1.724]	0.259^***^ *p* < 0.001; [0.219; 0.306]
**OPS-Code** comparison group 5-530.31 TAPP	35,281		
5-530.32 TEP	17,981	1.313^*^ *p* = 0.011; [1.066; 1.618]	1.249^***^ *p* < 0.001; [1.136; 1.374]
5-530.33 open approach with anterior placement of the mesh	16,443	0.800^⋅^ *p* = 0.088; [0.619; 1.032]	1.858^***^ *p* < 0.001; [1.706; 2.026]
5-530.1 open primary tissue repair without mesh (suture-based repair)	7261	1.482^*^ *p* = 0.010; [1.101; 1.995]	2.374^***^ *p* < 0.001; [2.090; 2.699]
5-530.34 open approach with posterior placement of the mesh	4044	0.967 *p* = 0.877; [0.633; 1.479]	1.965^***^ *p* < 0.001; [1.640; 2.356]
5-530.71 recurrence TAPP	3701	1.843^***^ *p* < 0.001; [1.327; 2.561]	1.348^***^ *p* < 0.001; [1.147; 1.574]
5-530.73 recurrence anterior approach	2244	4.021^***^ *p* < 0.001; [2.998; 5.396]	2.297^***^ *p* < 0.001; [1.960; 2.693]
5-530.72 recurrence TEP	1452	3.905^***^ *p* < 0.001; [2.736; 5.572]	1.260^⋅^ *p* = 0.071; [0.979; 1.622]
5-530.5 recurrence open surgical repair with prosthetic reinforcemnet	556	9.609^***^ *p* < 0.001; [6.543; 14.106]	2.985^***^ *p* < 0.001; [2.180; 4.088]
5-530.74 recurrence posterior approach	556	3.745^***^ *p* < 0.001; [2.172; 6.453]	2.028^***^ *p* < 0.001; [1.401; 2.939]
5-530.03 open without further action	357	4.273^***^ *p* < 0.001; [2.231; 8.189]	4.507^***^ *p* < 0.001; [3.360; 6.038]
**gender** comparison group male	81,208		
female	9304	1.045 *p* = 0.715; [0.826; 1.320]	1.015 *p* = 0.779; [0.919; 1.120]
age (years)		1.003 *p* = 0.323; [0.997; 1.009]	1.010^***^ *p* < 0.001; [1.008; 1.013]
obesity (E66)	11,702	1.716^***^ *p* = 0.002; [1.402; 2.100]	1.263^***^ *p* < 0.001; [1.144; 1.395]
COPD (J44)	9283	1.207 *p* = 0.155; [0.931; 1.565]	1.245^**^ *p* < 0.001; [1.144; 1.395]
Anticoagulation (Z92.1)	10,254	1.275. *p* = 0.052; [0.999; 1.628]	1.529^***^ *p* < 0.001; [1.389; 1.680]
diabetes (E10-E14)	12,809	1.312^*^ *p* = 0.019; [1.045; 1.648]	1.143^**^ *p* = 0.009; [1.028; 1.271]
dementia (F00-F03, G30)	2964	0.989 *p* = 0.958; [0.651; 1.504]	1.206^**^ *p* = 0.008; [1.049; 1.388]
visual impairment (H54.0-H54.2)	926	1.089 *p* = 0.802; [0.564; 2.101]	1.153 *p* = 0.267; [0.897; 1.461]
hearing loss (H90, H91)	18,515	1.133 *p* = 0.164; [0.949; 1.353]	1.057 *p* = 0.142; [0.981; 1.147]
urinary incontinence (R32, N39.3, N39.4)	4822	1.216 *p* = 0.225; [0.886; 1.670]	1.013 *p* = 0.844; [0.895; 1.151]
faecal incontinence (R15)	1256	0.708 *p* = 0.360; [0.341; 1.468]	1.044 *p* = 0.687; [0.837; 1.270]
need for care (Z74)	5869	0.880 *p* = 0.435; [0.639; 1.212]	1.150^*^ *p* = 0.013; [1.016; 1.300]
living alone /social isolation (Z60)	1547	0.962 *p* = 0.896; [0.541; 1.710]	1.266^*^ *p* = 0.022; [1.039; 1.543]
self-reported psychosocial stressors (Z91.8)	349	0.963 *p* = 0.949; [0.305; 3.042]	1,799^**^ *p* = 0.002; [1.249; 2.595]
**mobility restriction** comparison group no restriction– Barthel 100 points, FIM 85–91 points (U50)	87,033		
slight restriction – Barthel 80–95 points, FIM 69–84 points (U50.1)	1153	0.654 *p* = 0.270; [0.308; 1.389]	1.297^*^ *p* = 0.013; [1.053; 1.595]
moderate restriction – Barthel 60–75 points, FIM 59–68 points (U50.2)	648	0.817 *p* = 0.660; [0.333; 1.824]	1.096 *p* = 0.507; [0.826; 1.428]
severe restriction – Barthel ≤ 55 points, FIM ≤ 88 points (U50.3-U50.5)	1181	0.658 *p* = 0.299; [0.299; 1.445]	1.138 *p* = 0.220; [0.935; 1.413]

*p* < 0,1, * *p* < 0,05, ** *p* < 0,01, *** *p* < 0,001,

According to the allocation criteria explained at the beginning – specifically, contextual factors and ASA score – specific ICD codes were included as covariates. In addition, the Elixhauser score was tested as a sum measure in separate models, where the individual diagnoses of obesity, COPD, and diabetes were not included as separate covariates, as they are already included in the Elixhauser score. In the regression for the reoperation rate, the Elixhauser score showed no statistical significance (*p* = 0.557). In the regression analysis for postoperative complications, the score was statistically significant (*p* < 0.001). Still, with an odds ratio of 1.001, it showed an almost negligible effect size, which is why the selected individual diagnoses were used instead.

Both regressions with the individual diagnoses (Table 1) were significant according to the omnibus test; however, they explained only a tiny proportion of the variance (McFadden’s R-squared values of 0.033/0.041) and showed low discriminant power (AUC values of 0.646/0.659). There were apparent differences between the care sectors. Outpatient procedures were associated with an increased reoperation rate compared to inpatient care; however, they were also associated with a three- to four-times lower complication rate. Within the outpatient sector, operations in practices achieved better results than outpatient hospital treatments.

There were many differences in success rates between the various surgical methods. Compared to TAPP as a reference, other procedures for primary operations showed predominantly no or only marginally significant differences in terms of reoperation rates. Complications, on the other hand, occurred significantly more frequently with the different procedures. The respective procedure used for recurrent operations was always associated with poorer results.

In the model addressing reoperations, only a small number of variables were statistically significant. Obesity showed the most pronounced association with an increased likelihood of reoperation, whereas diabetes was associated with a more moderate effect size. Anticoagulation demonstrated a borderline association and should therefore be interpreted with caution.

In the model examining postoperative complications, a larger number of variables were statistically significant. Anticoagulation and self-reported psychosocial stressors exhibited comparatively higher odds ratios, whereas obesity, COPD, dementia, need for care, and mild mobility restrictions were associated with moderate increases in risk.

Additionally, interaction effects between the care sector and other patient- and procedure-related variables were examined with respect to surgical outcomes. At a significance level of 1%, only the interaction with female gender was statistically significant in the model assessing unsuccessful surgery; however, the associated odds ratio was very close to unity (OR = 1.02), indicating a negligible effect size. In the model for postoperative complications, three interaction terms were statistically significant at the 1% level. The interaction between recurrent TAPP procedures and outpatient treatment in a hospital setting showed a high odds ratio but was considered unstable due to the extremely wide confidence interval [2.95; 458.63]. The interaction between age and outpatient hospital care had a small effect, with an odds ratio of 0.98, whereas the combination of female gender and outpatient treatment in a physician’s office resulted in an odds ratio of 1.76. Importantly, when applying a more stringent significance threshold of 0.1%, none of the interaction effects remained statistically significant. Given the large number of interaction terms tested, these findings are therefore likely to reflect random variation rather than systematic, clinically meaningful sector-specific effects. Overall, the interaction analyses do not provide evidence to support differential outcome effects across care sectors.

## Discussion

In relation to the primary question of patient allocation to care sectors, the available data must first be contextualized. The rate of outpatient surgeries (17.1%) is in line with other studies from Germany for the same period [4, 6]. Reoperation (0.9%) and complication rates (4.8%) are also comparable, but vary depending on the parameters considered [18, 20, 21]. Despite consistency with other studies, the use of routine data from BKK-insured persons carries potential biases due to selection bias, coding quality, and economically motivated coding.

Many variables were considered in the regressions, but the proportion of explained variance remained very low. This means that the frequently discussed variables have only a minor influence on the success of the operation. In the case of reoperations, recurrence operations, surgical procedures, the care sector, obesity, and diabetes were significant influencing factors. Regarding complications, other diseases and social factors were also considered. Overall, in the context of sector allocation, the decisive question is whether the association between patient- and procedure-related risk factors and outcomes is modified by the care sector, warranting the examination of interaction effects. Based on these analyses, there is no reliable evidence of a systematic, sector-specific effect that would justify targeted allocation.

Contrary effects are evident for the desired shift to outpatient care, with inpatient operations being associated with fewer reoperations and more complications. Given the low rate of 0.9% reoperations, the absolute effect size is of negligible practical significance, despite the increased odds ratio. In the context of the overall weak variance explanation, the care sector’s influence on the operation’s success remains correspondingly limited.

The suitability of contextual factors or ASA scores for sector allocation appears questionable based on the data. In younger adults, relevant contextual factors are likely to be rare in the common disease of inguinal hernia, yet treatment is essentially inpatient. There is no apparent medical justification for this extent at the aggregate level – especially since the outpatient share is significantly higher in children and adolescents.

In line with the absence of robust interaction effects and the limited explanatory value of contextual factors and ASA-related characteristics in the present analyses, inpatient treatment cannot be preferentially justified at the aggregate level for primary inguinal hernia repair. While inpatient care may be appropriate in selected individual cases—for example, when outpatient management is not feasible for practical or organizational reasons or when clinically relevant complications are anticipated—the data do not indicate general medical quality-related objections to outpatient treatment. Given the low rates of reoperation and complications, inpatient care should be regarded as an option for exceptional circumstances rather than a default strategy. Consequently, outpatient treatment may be pursued whenever feasible, particularly considering economic considerations, while preserving individual clinical judgment in such exceptional cases. Within this framework, the introduction of hybrid DRGs introduces a sector-independent reimbursement scheme that economically favors outpatient treatment.

Beyond patient-related predictors of sector allocation, the analysis showed that the choice of surgical procedures was largely invariant across care sectors and patient ages. The literature offers mixed evaluations of surgical techniques, ranging from broad equivalence to reported advantages of laparoscopic approaches such as TAPP and TEP [22, 23]. The present analysis aligns with studies reporting more favorable outcomes for laparoscopic procedures.

Laparoscopic procedures are associated with higher direct costs for service providers, for example, due to increased material costs. With the introduction of hybrid DRGs, cross-sector flat-rate remuneration is now in place, regardless of the procedure chosen. For example, a non-complex unilateral inguinal hernia repair (hybrid DRG G24M) is reimbursed at €1,852.71 in 2025, irrespective of whether the procedure is performed on an outpatient or inpatient basis [24]. This can create selection pressure, which is likely to increase cost pressure on the choice of procedure during outpatient treatment. This assumption is also shared by other analyses [2]. This can be detrimental to the economy as a whole because indirect costs rise due to more extended convalescence and increased absences from work. Some studies, therefore, consider laparoscopic procedures to be more economically efficient [25, 26].

As initially planned, a hybrid DRG was to be calculated on a cost basis – analogous to the G-DRG system – so that different costs would be reflected in the hybrid DRG depending on the procedure chosen. If the costs of the other procedures differed significantly, this would result in a hybrid DRG split, which would then create adequate remuneration.

## Conclusion

Only a small portion of surgical success can be attributed to common predictors, and no interaction effects with the care sector were observed. These results suggest that sector allocation does not determine outcomes, emphasizing the ongoing potential for outpatient inguinal hernia surgery. Remuneration models should support outpatient care while accounting for procedure-specific costs to preserve clinical freedom and improve patient outcomes. Future studies should assess how the introduction of hybrid DRGs affects sector allocation by comparing post-reform patterns with the baseline presented in this analysis. Further research should clarify cost differences between surgical procedures.
